# Food allergy-related concerns during the transition to self-management

**DOI:** 10.1186/s13223-019-0370-1

**Published:** 2019-09-05

**Authors:** Jennifer Lisa Penner Protudjer, Roelinde Middelveld, Sven-Erik Dahlén, Staffan Ahlstedt, FoodHE Investigators, Sven-Arne Jansson, Eva Östblom, Marianne Heibert Arnlind, Ulf Bengtsson, Ingrid Kallström-Bengtsson, Birgitta Marklund, Georgios Rentzos, Ann-Charlotte Sundqvist, Johanna Åkerström

**Affiliations:** 1 Institute of Environmental Medicine, Karolinska Institutet, Stockholm, Sweden; 2 Department of Pediatrics and Child Health, University of Manitoba, Winnipeg, Canada; 3 George and Fay Yee Centre for Healthcare Innovation, Winnipeg, Canada; 4 The Children’s Hospital Research Institute of Manitoba, Winnipeg, Canada; 5 The Centre for Allergy Research, Karolinska Institutet, Stockholm, Sweden

**Keywords:** Age, Food allergy, Gender, Health-related quality of life

## Abstract

**Background:**

Compared to non-allergic individuals, food allergic individuals have impaired health-related quality of life (HRQL). However, effects of gender and age are unclear. The objective of our study was to describe associations between allergies to common foods and HRQL with consideration to gender and age.

**Methods:**

Adolescents and adults (N = 137; 49.6% males) with specialist-diagnosed allergy to milk, egg and/or wheat completed age-appropriate versions of the Food Allergy Quality of Life Questionnaire (FAQLQ). We pooled common questions and calculated overall- and domain-specific HRQL in association with number and severity of symptoms and time elapse since worst reaction.

**Results:**

In the entire study population, HRQL was not affected by gender or age, whereas gender-specific age categories affected HRQL among males only. For example, males 18–39 years had worse overall- (β = 0.77; 95% CI 0.08–1.45) and domain-specific HRQL vs. males < 18 years. Among participants with 1–3 food allergy symptoms, no associations were found. Among participants with 4–6 symptoms, the domain allergen avoidance and dietary restrictions was worse among older participants (e.g. 40+ years: β = 0.71; 95% CI 0.05–1.37 vs. < 18 years), and males 18–39 vs. < 18 years. Among participants with severe symptoms, females vs. males, and participants 18–39 vs. < 18 years had worse HRQL. At least 4 years since worst reaction was associated with worse HRQL for participants 40+ years vs. < 18 years, and older males vs. males < 18 years. Nearly all differences exceeded the clinical relevance threshold of ≥ 0.5.

**Conclusions:**

Associations between allergies to common foods and HRQL are affected by gender and age. Most affected are males 18–39 years. Among females, HRQL is more stable across age groups.

## Background

Food allergy affects as many as 10% of people worldwide, with children and youth disproportionately represented [1, 2]. Moreover, most who have food allergy, particularly to peanuts and tree nuts, in early life will experience disease persistence [2, 3]. Individuals living with a food allergy are counselled to avoid the food(s) to which they are allergic, in order to minimize the risk of adverse reactions [1]. At the same time, they are encouraged to always carry an epinephrine autoinjector, for use in the event of an allergic reaction. The most severe food allergic reaction is anaphylaxis, a multi-system reaction that mandates emergency treatment and is potentially fatal [4, 5]. Although anaphylaxis is rare [5, 6], nearly half of young people with food allergy report previous, less severe allergic reactions [6].

Given the requisite dietary and behavioural changes necessitated by food allergy, and the ever-present risk for an allergic reaction, it is unsurprising that food impacts health-related quality of life (HRQL) [7–11]. Moreover, as gender seems to impact health beliefs and the evaluation of potentially risky food-related situations [12], it is also unsurprising that, amongst those with food allergy, females report worse HRQL than males [11]. Compared to males, females are less likely to engage in risk behaviours [13], better cope with the transition to self-management [14], but have more food allergy-related anxiety [4].

Similarly, differences are seen at various developmental stages [9, 10]. Development and coping are strongly interconnected, and influenced by interpersonal meaning and context [15]. Many young people with food allergy search for a way to integrate their disease within their identity, whereas others will reject it [10]. Yet, the ability of young people to adapt to their chronic disease strongly predicts favourable long term coping [15]. As such, it is critical that healthcare professionals are able to identify coping, and thus self-management abilities, amongst their food allergic patients, in order to minimise patients’ risk for allergic reactions and help them maximise their health-related quality of life.

Even less is known about the interaction of gender and age on self-management, coping and HRQL amongst those with food allergy. Yet, as food allergy is more prevalent in young people than adults [1], young people are more likely to be in need of healthcare. But, as many of these young people are likely to experience persistent disease [2, 3], it behoves healthcare professionals to gain insight into the coping mechanisms and reported challenges of this age group. Thus, the aim of this study was to examine associations between allergies to common foods, including severity, and different aspects of HRQL with consideration to gender and age.

## Methods

### Patients and procedures

Patient selection has been described elsewhere in detail [7, 8]. Briefly, in 2010–2011, Swedish adolescents (13–17 years old) and adults (18+ years old) with specialist-diagnosed allergy to the common foods cow’s milk, hen’s egg and/or wheat, all staples in the Swedish diet, were identified and recruited from outpatient allergy clinics in the Swedish cities of Stockholm and Gothenburg, respectively. Inclusion criteria were a convincing history of allergy to at least one common food, ascertained either by a positive food challenge, or by high levels of food specific Immunoglobulin E (IgE) antibodies associated with a 95% probability of a positive result in a double-blind placebo controlled food challenge in combination with a convincing clinical history to the same food [16, 17]. Adolescents and adults completed the Food Allergy Quality of Life Teenager Form (FAQLQ-TF) and Adult Form (FAQLQ-AF), respectively. We made use of the same data as used in previous publications [7, 8]. However, the analyses performed herein do not overlap any of those reported previously.

### Food Allergy Quality of Life Questionnaire Teenager Form (FAQLQ-TF) and Adult Form (FAQLQ-AF)

The development of food allergy-specific HRQL instruments, such as the Food Allergy Quality of Life Questionnaires (FAQLQ), developed by EuroPrevall [18, 19], and previously used in our studies [7, 8, 20], has been demonstrated to permit identification of issues unique to those living with food allergies. The FAQLQ have been validated elsewhere and permit the opportunity to discriminate between different food allergy characteristics [18, 19]. Questions common to both the FAQLQ-TF and FAQLQ-AF (61%; 14/23; Additional file 1: Table S1) were included. Responses to these questions were on scale from 1 = best HRQL to 7 = worst HRQL, and were merged into a single data set, from which we calculated overall- and domain-specific HRQL scores. Described in Additional file 1: Table S2, these domains were allergen avoidance and dietary restrictions (AADR) (8/10 [80%] common questions); emotional impact (EI) (4/7 [57%] common questions) and risk of accidental exposure (RAE) (2/6 [33%] common questions). Of particular interest was AADR, which reflects challenges associated with food allergy-related self-management.

We excluded one participant (an adolescent) who did not provide a birthdate. For the two adults who only provided year of birth, we randomly assigned birthdates of 2 July, representing the mid-date of a calendar year. Age was further categorised into three categories: < 18 years; 18–39 years; and 40+ years, to permit approximately similar size groups and to approximately align with other studies on transition to self-management [21].

### Definitions of variables

*Self*-*reported symptoms* were classified as oral (itchy mouth, tongue or lips; swollen tongue or lips), rhinoconjunctivitis (runny or blocked nose; itchy or watery eyes), dermatological (itchy skin, rash, oedema or hives), gastrointestinal (nausea; vomiting; cramping; diarrhoea), lower respiratory (swollen throat; difficulty swallowing; hoarseness; wheeze; or, shortness of breath) or cardiovascular/neurological (tachycardia; loss of vision; unable to stand; lightheadedness). As shown in Additional file 1: Table S3, symptoms were subsequently grouped as less severe (oral; dermatological; gastrointestinal) or more severe (rhinoconjunctivitis; lower respiratory; cardiovascular/neurological).

*Time since most severe reaction* ranged from 1 week to 20 years, and was collapsed into a binary variable, at the median of 4 years. Participants self-reported epinephrine auto-injector (EAI) prescriptions.

*The number of common food allergies* was categorised in two mutually exclusive groups of one, or two to three allergies to common foods. Time elapse since most severe reaction ranged from 1 week to 20 years. For the two adolescents who reported that ‘several years’ had elapsed since their last reaction, we randomly assigned a time of 5 years for statistical analysis purposes. Time elapse was considered as a binary variable, divided at the median of 4 years. Participants self-reported if they had been prescribed an epinephrine auto-injector (EAI).

### Statistical analysis

Descriptive statistics included sample sizes (n), means, standard deviations, percentages and 95th percent confidence intervals (95% CI). With the exception of the background characteristics, all analyses are presented by gender and age group (< 18 years; 18–39 years; 40+ years). As our aim was to explore gender and age in relation to HRQL, we first analysed data with consideration to gender, then to age, and subsequently to gender-specific age categories.

Linear regression was used to test for differences in overall- and domain-specific HRQL scores between various groups, for which corresponding β co-efficients and 95% CI are reported. Potential confounders were considered based on a priori knowledge. The final model included adjustments for the number of allergies to any food, and EAI prescription. Given that peanuts and tree nuts were the most commonly offending non-common food, we performed a sensitivity analysis, in which we additionally included this variable as a confounder. This additional adjustment insubstantially altered (< 10%) most point estimates, and did not statistically significantly alter any of the point estimates. As such, the results are not presented herein.

Statistical significance was set at p < 0.05. Statistical analyses were performed with Stata 13.1 for Windows (College Station, TX USA). In keeping with other publications [18, 22], including one from our group [8], we contextualized statistical significance with clinically relevant HRQL, set at a minimal clinically important difference (MCID) of ≥ 0.5 of HRQL scores for a given comparison. As many statistically significant differences were also clinically relevant, we highlight statistically significant differences which do not reach an MCID of ≥ 0.5.

Peanuts and tree nuts were the most common non-common food allergy, but neither substantially nor significantly altered any point estimates in a sensitivity analysis.

### Ethics

This study was approved by the Regional Ethical Review Board in Stockholm, Sweden (Dnr 2009/84-31/5). Personal data were treated according to the Swedish Personal Data Act.

## Results

Overall, 137 (n = 68; 49.6% males) participants were included (Table 1). Males were younger than females (24.8 vs. 33.5 years; p < 0.01). Both genders averaged 1.5 allergies to common foods, had similar numbers of allergies to other foods, and EAI prescriptions. Overall- and domain specific HRQL were comparable between the genders, with the exception of EI, which was worse amongst females than males (Table 2). For both genders combined, compared to < 18 years, 18–39 years had worse AADR, whereas 40+ years had significantly worse overall HRQL and EI, but which did not quite reach the MCID threshold (e.g. EI for 40+ years: ß = 0.46; 95% CI 0.01–0.91).

**Table 1:** Background characteristics of the study population, by gender

	Males (n = 68)			Females (n = 69)		
	n	Mean	95% CI	n	Mean	95% CI
Age (years)	68	24.8	12.7; 59.4	69	33.5	13.5; 64.8
Average number of allergies to common foods	68	1.5	1; 3	69	1.5	1; 3
Average number of allergies to other foods^a^	68	1.6	0; 4	69	1.7	0; 4
Time since worst reaction (years)	65	5.3	0.4; 15	59	5.0	0.2; 15

	Males (n = 68)			Females (n = 69)		
	n	Percent	95% CI	n	Percent	95% CI
By age category (years)						
< 18	39	57.4	44.8; 69.3	18	26.1	16.3; 38.1
18–39	16	23.5	14.1; 35.4	25	36.2	25.0; 48.7
≥ 40	13	19.1	10.6; 30.5	26	37.7	26.3; 50.2
Allergies to common foods^b^						
Cow’s milk	40	58.8	46.2; 70.6	35	50.7	38.4; 63.0
Hen’s egg	43	63.2	50.7; 74.6	42	60.9	48.3; 72.4
Wheat	20	29.4	19.0; 41.7	25	36.2	25.0; 48.7
Allergies to other foods^a^						
Peanuts/tree nuts	44	64.7	52.2; 75.9	45	65.2	52.8; 76.3
Fish/shellfish	23	33.8	22.8; 46.2	23	33.3	22.4; 45.7
Soy	11	16.2	8.4; 27.1	13	18.8	10.4; 30.1
Sesame	7	10.3	4.2; 20.1	3	4.3	0.9; 12.2
Fruits/vegetables	26	38.2	26.7; 50.8	31	44.9	32.9; 57.4
Self-reported symptoms^b^						
Oral	66	97.1	89.8; 99.6	66	95.7	87.8; 99.1
Rhinoconjunctivitis	38	55.9	43.3; 67.9	38	55.1	42.6; 67.1
Dermatological	45	55.9	43.3; 67.9	45	55.1	42.6; 67.1
Gastrointestinal	41	60.3	47.7; 72.0	56	81.2	69.9; 89.6
Lower respiratory	52	76.5	64.6; 85.9	51	73.9	61.9; 83.7
Cardiovascular/neurological	24	35.3	24.1; 47.8	26	37.7	26.3; 50.2
Epinephrine auto-injector prescription	47	69.1	56.7; 79.8	37	53.6	41.2; 65.7

^a^Includes participant-perceived or doctor-diagnosed allergies to other foods

^b^Non-mutually exclusive categories. Percentages reflect the proportion of respondents with a particular characteristic (type of food allergy, symptom) in relation to the total number of males/females. As such, column percentages exceed 100%

**Table 2:** Associations between gender- and age categories and total- and domain specific health-related quality of life (HRQL)

	Overall HRQL			AADR			EI			RAE		
	Mean	β^a^	95% CI	Mean	β^a^	95% CI	Mean	β^a^	95% CI	Mean	β^a^	95% CI
Gender												
Males	5.00	Referent		5.43	Referent		4.49	Referent		4.34	Referent	
Females	5.23	0.33	− 0.05; 0.69	5.63	0.27	− 0.13; 0.63	*4.78* ^†^	*0.44*	*− 0.01; 0.89*	4.58	0.30	− 0.31; 0.91
Age categories (years)												
< 18	4.96	Referent		5.25	Referent		4.65	Referent		4.45	Referent	
18–39	5.34	0.17	− 0.06; 0.39	*5.81* ^†^	*0.27*	*0.03; 0.41*	4.84	0.01	− 0.27; 0.28	4.46	0.10	− 0.28; 0.48
40+	*5.11* ^†^	*0.38*	*0.01; 0.75*	5.65	0.33	− 0.06; 0.72	*4.40* ^†^	*0.46*	*0.01; 0.91*	4.47	0.47	− 0.14; 1.08
Age-specific gender categories												
< 18 years												
Males	4.81	Referent		5.14	Referent		*4.35*	Referent		4.46	Referent	
Females	5.29	0.52	− 0.11; 1.15	5.49	0.41	− 0.25; 1.07	*5.30* ^‡^	*1.00*	*0.26; 1.75*	4.42	− 0.02	− 1.05; 1.00
18–39 years												
Males	5.63	Referent		6.12	Referent		5.11	Referent		4.75	Referent	
Females	5.15	− 0.30	− 1.07; 0.47	5.61	− 0.37	− 1.15; 0.41	4.68	− 0.16	− 1.01; 0.69	4.28	− 0.29	− 1.65; 1.06
40+ years												
Males	4.80	Referent		5.48	Referent		4.15	Referent		*3.46*	Referent	
Females	5.27	0.47	− 0.18; 1.13	5.76	0.44	− 0.30; 1.18	4.52	0.21	− 0.69; 1.11	*4.98* ^‡^	*1.19*	*0.18; 2.20*

Italic denotes statistically significant results

HRQL is on a scale from 1 to 7, where 1 = best possible HRQL, and 7 = worst possible HRQL

*HRQL* health-related quality of life, *AADR* allergen avoidance and dietary restrictions, *EI* emotional impact, *RAE* risk of accidental exposure

^†^p < 0.05

^‡^p < 0.01

^a^Represent mean difference in outcome compared to the reference group. Calculated by linear regression, adjusted for number of food allergies and prescription of an epinephrine autoinjector

Compared to males < 18 years, those 18–39 years had significantly worse HRQL (e.g. overall: β = 0.73; 95% CI 0.07–1.39; AADR: β = 0.93; 95% CI 0.23–1.62; Table 3). Amongst females, mean HRQL scores varied little and non-significantly between age groups.

**Table 3:** Associations between gender-specific age categories and total- and domain specific health-related quality of life (HRQL), in the entire study population

	Overall HRQL			AADR			EI			RAE		
	Mean	β^a^	95% CI	Mean	β^a^	95% CI	Mean	β^a^	95% CI	Mean	β^a^	95% CI
Males (years)												
< 18	4.81	Referent		5.14	Referent		4.35	Referent		4.46	Referent	
18–39	*5.63* ^†^	*0.73*	*0.07; 1.39*	*6.12* ^‡^	*0.93*	*0.23; 1.62*	5.11	0.64	− 0.15; 1.43	4.75	0.14	− 1.01; 1.28
40+	4.80	0.18	− 0.58; 0.93	5.48	0.43	− 0.36; 1.21	4.15	0.13	− 0.77; 1.04	3.46	− 0.66	− 1.96; 0.64
Females (years)												
< 18	5.29	Referent		5.49	Referent		5.31	Referent		4.42	Referent	
18–39	5.15	0.33	− 0.82; 0.51	5.61	0.10	− 0.61; 0.80	4.68	− 0.65	− 1.45; 0.15	4.28	− 0.19	− 1.25; 0.86
40+	5.27	0.31	− 0.03; 0.39	5.76	0.33	− 0.34; 1.00	4.52	− 0.63	− 1.40; 0.13	4.98	0.63	− 0.37; 1.63

Italic denotes statistically significant results

HRQL is on a scale from 1 to 7, where 1 = best possible HRQL, and 7 = worst possible HRQL

*HRQL* health-related quality of life, *AADR* allergen avoidance and dietary restrictions, *EI* emotional impact, *RAE* risk of accidental exposure

^†^p < 0.05

^‡^p < 0.01

^a^Represent mean difference in outcome compared to the reference group. Calculated by linear regression, adjusted for number of food allergies and prescription of an epinephrine autoinjector

Consideration to less severe symptoms in association with HRQL was not possible as too few participants had only less severe symptoms. Amongst those with more severe symptoms, males 18–39 years vs. < 18 years had worse AADR, the domain most reflective of self-management (β = 0.80; 95% CI 0.01–1.59; Table 4).

**Table 4:** Associations between gender-specific age categories and total- and domain specific health-related quality of life (HRQL), with consideration to severe symptoms, and time since worst reaction

	Overall HRQL			AADR			EI			RAE		
	Mean	β^a^	95% CI	Mean	β^a^	95% CI	Mean	β^a^	95% CI	Mean	β^a^	95% CI
Severe symptoms												
Males (years)												
< 18	4.82	Referent		5.14	Referent		4.39	Referent		4.38	Referent	
18–39	5.69	0.62	− 0.15; 1.38	*6.15* ^†^	*0.80*	*0.01; 1.59*	5.25	0.61	− 0.27; 1.50	4.71	− 0.11	− 1.40; 1.17
40+	4.94	0.24	− 0.64; 1.12	5.43	0.36	− 0.56; 1.27	4.65	0.40	− 0.62; 1.43	3.55	− 0.49	− 1.97; 1.00
Females (years)												
< 18	5.56	Referent		5.75	Referent		5.63	Referent		4.68	Referent	
18–39	5.19	− 0.34	− 1.05; 0.38	5.64	− 0.11	− 0.89; 0.67	*4.69* ^†^	*− 0.87*	*− 1.75, 0.00*	4.45	− 0.21	− 1.39; 0.96
40+	5.39	− 0.19	− 0.90; 0.52	5.84	0.01	− 0.77; 0.79	4.67	− 0.96	− 1.83; − 0.09	5.28	0.66	− 0.50; 1.82
0–3 years since worst reaction												
Males (years)												
< 18	5.18	Referent		5.52	Referent		4.54	Referent		5.13	Referent	
18–39	*6.40* ^‡^	*1.31*	*0.33; 2.30*	*6.85* ^†^	*1.39*	*0.24; 2.55*	*6.05* ^†^	*1.61*	*0.46; 2.76*	5.30	0.38	− 1.25; 2.00
40+	4.98	− 0.08	− 1.11; 0.94	5.60	0.08	− 1.12; 1.28	4.58	0.36	− 0.83; 1.55	3.33	− 1.55	− 3.24; 0.14
Females (years)												
< 18	5.62	Referent		5.80	Referent		5.73	Referent		4.70	Referent	
18–39	4.54	− 0.11	− 0.82; 0.60	5.91	0.12	− 0.66; 0.91	4.96	− 0.63	− 1.43; 0.16	4.50	− 0.02	− 1.43; 1.40
40+	5.30	− 0.20	− 0.92; 0.52	5.83	0.78	− 0.72; 0.87	*4.52* ^†^	*− 0.97*	*− 1.78; − 1.67*	4.75	0.22	− 1.21; 1.66
4+ years since worst reaction												
Males (years)												
< 18	4.23	Referent		4.53	Referent		4.05	Referent		3.40	Referent	
18–39	5.36	0.89	− 0.04; 1.82	*5.84* ^‡^	*1.28*	*0.36; 2.19*	4.85	0.35	− 0.90; 1.61	4.50	0.43	− 1.22; 2.08
40+	4.68	1.13	− 0.39; 2.65	*5.39* ^†^	*1.75*	*0.26; 3.24*	3.60	− 0.08	− 2.12; 1.95	4.10	1.22	− 1.47; 3.91
Females (years)												
< 18	4.89	Referent		5.29	Referent		4.61	Referent		3.86	Referent	
18–39	4.94	− 0.37	− 1.61; 0.88	5.40	− 0.23	− 1.61; 1.16	4.47	− 0.63	− 2.17; 0.90	4.11	− 0.33	− 2.14; 1.48
40+	5.54	0.29	− 0.91; 1.49	6.03	0.42	− 0.94; 1.78	4.74	− 0.32	− 1.80; 1.16	5.59	1.39	− 0.36; 3.13

Italic denotes statistically significant results

HRQL is on a scale from 1 to 7, where 1 = best possible HRQL, and 7 = worst possible HRQL

*HRQL* health-related quality of life, *AADR* allergen avoidance and dietary restrictions, *EI* emotional impact, *RAE* risk of accidental exposure

^†^p < 0.05

^‡^p < 0.01

^a^Represent mean difference in outcome compared to the reference group. Calculated by linear regression, adjusted for number of food allergies and prescription of an epinephrine autoinjector

Amongst those with 0–3 years since worst reaction and HRQL (Table 3), older males consistently had worse HRQL overall and across domains (e.g. overall HRQL: males 18–39 years vs. < 18 years: β = 1.31; 95% CI 0.33–2.30). Amongst females, only EI was statistically different: Women 40 + had *better* EI than women < 18 years (β = − 0.97; 95% CI − 1.78 to − 1.67).

Amongst males with 4+ years since worst reaction, older males, vs. < 18 years, had significantly worse overall AADR (18–39 years: β = 1.28; 95% CI 0.36–2.19; 40+ years β = 1.75; 95% CI 0.26–3.24). No associations were found for females, suggesting that food allergy has a strong impact on females across age categories, and which does not disappear with either age or time since worst reaction.

## Discussion

In this study of adolescents and adults allergic to common foods, we found differences in HRQL between genders and across age groups. Most affected are males 18–39 years, particularly within the domain, allergen avoidance and dietary restrictions, which captures the ability to manage social situations and cope with related troubles. Moreover, worse AADR may reflect heightened vulnerability, juxtaposed against limited adaptive behaviours and actions necessary for understanding risk and compliance [10]. In contrast, females reported more stable HRQL across age groups, suggesting a more consistent ability across developmental stages to cope and self-manage.

Self-management is the current cornerstone for food allergy healthcare providers. The transition to food allergy self-management is complex and difficult to master. Whereas a survey of United States-based pediatric allergists indicated that, by age 12–14 years, food allergic patients should start to share responsibility for the management of EAI use and anaphylaxis recognition [23], our data indicate that the ability to cope and manage may impact HRQL for a much longer period than previously posited. Thus, acknowledging differing food allergy-specific HRQL between age groups and genders is important for healthcare professionals as they guide their patients toward self-management. Although adolescent patients transition from pediatric care to adult care, healthcare professionals may play a critical role in the continuity of care, through ongoing patient contact, support groups and monitoring. Such continuity of care is particularly important for males in the early years in the transition to adult care. Between adolescence and the third decade of life, males decrease their risk taking behaviours and cognitively mature [13]. During this period, males are also likely to take more responsibility for food preparation. These behaviours may be considered two proxies for self-management.

For both time categories, older males consistently reported worse AADR than males < 18 years. These differences may be attributable to struggles to normalize food allergy over a longer period, juxtaposed against distant memories of a severe reaction. More broadly, older men may wish to normalise their food allergy by seeking control and integrating with others, yet recognise, as a result of maturity, that such actions may increase their risk of food allergic reaction.

We included only questions common to both the FAQLQ-TF and FAQLQ-AF. Nonetheless, this is one of the few HRQL studies on food allergy in which associations between gender and age are considered. In particular, we considered proxies for learning to cope with self-management.

## Conclusions

Associations between allergies to common foods, including food allergy severity, and HRQL are influenced by gender and age. Females had stable, but poor HRQL across age categories, whereas important differences exist across age categories amongst males. Most influenced are men 18–39 years, particularly within the domain, AADR, which encompasses self-management. These differences may be a proxy for how the genders cope with transition to food allergy self-management.

## Supplementary information

**Additional file 1.** Additional tables.

## Data Availability

The datasets analysed in the current study are not publicly available due to the sensitive nature of data collected from minor children.

## Abbreviations

AADR: allergen avoidance and dietary restriction
EAI: epinephrine autoinjector
EI: emotional impact
FAQLQ-AF: Food Allergy Quality of Life Questionnaire-Adult Form
FAQLQ-TF: Food Allergy Quality of Life Questionnaire-Teenager Form
HRQL: health-related quality of life
IgE: immunoglobulin E
MCID: minimal clinically important difference
RAE: risk of accidental exposure
95% CI: 95th percent confidence interval
