# Supplementary material for: Food allergy-related concerns during the transition to self-management
Source: Allergy Asthma Clin Immunol. 2019 Sep 5;15:54. doi: 10.1186/s13223-019-0370-1 (PMC6727333; doi:10.1186/s13223-019-0370-1)
Supplement: Supplementary file 1 — Additional file 1. Additional tables. [file 13223_2019_370_MOESM1_ESM.docx]

| **Table S1.** Questions common to both the Food Allergy Quality of Life (FAQLQ) - Adult Form and FAQLQ - Teen Form* | | | | | | | |
| --- | --- | --- | --- | --- | --- | --- | --- |
|  |  |  |  |  |  |  |  |
| **Domain 1. Allergen avoidance and dietary restrictions** | | | | | |  |  |
| *How troublesome do you find it, because of your food allergy, that you :* | | | | | | |  |
| Must always be alert to what you are eating? | | | | |  |  |  |
| Are able to eat fewer products? | | | |  |  |  |  |
| Are limited to the products that you can buy? | | | | |  |  |  |
| Must read labels? | |  |  |  |  |  |  |
| Are less able to spontaneously accept an invitation to stay for a meal? | | | | | | |  |
| Are less able to taste or try various products when eating out? | | | | | | |  |
| Must always check yourself whether you can eat something when eating out? | | | | | | | |
| Hesitate eating a product when you have doubts about it? | | | | | |  |  |
|  |  |  |  |  |  |  |  |
| **Domain 2. Emotional Impact** | | |  |  |  |  |  |
| How discouraged do you feel during an allergic reaction? | | | | | |  |  |
| *How troublesome do you find it, because of your food allergy, that you :* | | | | | | |  |
| Have the feeling that you have less control of what you eat when eating out? | | | | | | | |
| *How frightened are you because of your food allergy:* | | | | | |  |  |
| Of an allergic reaction? | | |  |  |  |  |  |
| Of accidentally eating something wrong? | | | | |  |  |  |
|  |  |  |  |  |  |  |  |
| **Domain 3. Risk of Accidental Exposure** | | | |  |  |  |  |
| *How troublesome is it, because of your food allergy:* | | | | |  |  |  |
| That the ingredients of a food change? | | | |  |  |  |  |
| That the label states, "May contain (traces of)…? | | | | |  |  |  |
|  |  |  |  |  |  |  |  |
| HRQL is on a scale from 1-7, where 1=best possible HRQL, and 7= worst possible HRQL | | | | | | | |

**Table S2.** Descriptions of food allergy-specific health-related quality of life (HRQL), overall and by domains, as ascertained by food allergy-specific questionnaires

| **HRQL Element** | **Description** |
| --- | --- |
| Overall HRQL | Mean score of all three HRQL domains (AADR, EI, RAE) |
| AADR | Perceived trouble, due to food allergy, determining which foods can be safely consumed and how social situations can be managed |
| EI | Discouragement, disappointment, lack of control and fear, due to food allergy |
| RAE | Perceived trouble, due to food allergy, explaining food allergy to others, not being able to eat what others eat in social situations and that labelling may change/indicates 'may contain (traces of)' |

HRQL: Health-related Quality of Life; AADR: Allergen Avoidance and Dietary Restrictions; EI: Emotional Impact; RAE: Risk of Accidental Exposure

**Table S3.** Self-reported symptoms, classified by system and severity

| Symptoms | System | Severity |
| --- | --- | --- |
| Itchy mouth, tongue, lips | Oral | Less severe |
| Swollen tongue, lips |  |  |
| Runny or blocked nose | Rhinoconjuctivitis |  |
| Itchy or watery eyes |  |  |
| Itchy skin, rash | Skin |  |
| Oedema, hives |  |  |
| Nausea, vomiting | Gastrointestinal |  |
| Cramping, diarrhoea |  |  |
| Swollen throat, difficulty swallowing | Respiratory | More severe |
| Hoarseness, wheeze |  |  |
| Shortness of breath |  |  |
| Tachycardia | Cardiovascular/neurological |  |
| Loss of vision |  |  |
| Unable to stand |  |  |
| Lightheadedness |  |  |
